# Regional Interplay for Temporal Processing in Parkinson’s Disease: Possibilities and Challenges

**DOI:** 10.3389/fneur.2015.00270

**Published:** 2016-01-18

**Authors:** Michael Schwartze, Sonja A. Kotz

**Affiliations:** ^1^Department of Neuropsychology and Psychopharmacology, Faculty of Psychology and Neuroscience, Maastricht University, Maastricht, Netherlands; ^2^Department of Neuropsychology, Max Planck Institute for Human Cognitive and Brain Sciences, Leipzig, Germany

**Keywords:** Parkinson’s disease, temporal processing, timing, compensation, network

## Abstract

Parkinson’s disease (PD) is primarily associated with two dominant features: cardinal motor symptoms and the loss of cells in the substantia nigra pars compacta of the basal ganglia. Consequently, these aspects are major foci in PD-related research. However, PD is a neurodegenerative disease, which progressively affects multiple brain regions outside the basal ganglia and leads to symptoms outside the motor domain. Much less is known about the individual contribution of these secondary regions, their interplay and interaction with the basal ganglia, and the respective network dynamics in the overall manifestation of PD. These regions include classical motor structures such as the cerebellum and the supplementary motor area (SMA). However, just as the basal ganglia, these regions display a fine-grained microarchitecture, which supports sensory and sensorimotor functions. One such function is temporal processing, which has been ascribed to a network comprising all of these regions. On the one hand, pathological changes in this temporal processing network may be part and parcel of motor and non-motor symptoms in PD. On the other hand, a better understanding of the role of each network node may offer a novel perspective on compensatory mechanisms, therapeutic interventions, as well as the heterogeneity and individual differences associated with PD. We unfold this perspective by relating the neural foundations and functional implications of temporal processing to pathophysiological and neurofunctional changes characteristic of PD.

## Introduction

The basal ganglia system is a complex of several nuclei deeply embedded into the vertebrate brain. It has extensive connections to other subcortical and cortical regions, and via these connections, the basal ganglia system contributes to a wide range of motor and non-motor behaviors (1–5). One of the most fundamental functions attributed to the basal ganglia and associated brain regions is the patterning and chunking of behavior from simple motion sequences to complex cognitive sequences (6, 7). Motor patterns are expressed in physical motion, whereas cognitive patterns are not. They may be conceived as routines or “habits of thought,” reflecting a dominant processing mode, which emerges from the repetitive future-oriented sequencing of cognitive actions (6). These mechanisms reflect a common theme across behavioral domains, and likewise, a general basal ganglia function, which may guide both the iterative build-up and the evaluation of repetitive motor and cognitive behaviors (8).

In this context, the term “sequencing” may be broadly characterized as a function that pertains to the sequential order of motor and cognitive actions as well as to the specific temporal structure of these sequences. In other words, sequencing not only defines the order of successive actions in time but also their specific temporal relations. Motor and cognitive patterns are typically expressed as entire sequences of actions, i.e., they are “packaged as a unit ready for expression,” and monkey research indicates that accented neural activity marks the boundaries of such packages (8, 9). These accentuations introduce a temporal non-linearity, as “time can be decoded with higher resolution at the beginning and the end of the movement sequences than during them” (8). Considering that motor and cognitive patterns may not only be expressed in isolation, the continuous chunking of actions may effectively give rise to a combination of slower and faster dynamics in ongoing sequential behavior. Although it may be difficult to explicitly decode the temporal fine structure of actions that constitute the fast dynamics, it may be possible and functionally relevant to track the temporal structure of accented boundary markers, which constitute the slow dynamics. It has been suggested that the alternation of accentuation and de-accentuation reflects low attentional demands required to process actions between the markers (8). The questions arise if such temporal non-linearities have a perceptual equivalent, and if they can be exploited to optimize the allocation of attention in time for both, the production and the perception sequential behavior (10).

Habitual behaviors unfold in the hundreds-of-milliseconds to seconds range that is central to research into interval timing, another fundamental behavioral function that has been ascribed to the basal ganglia system, and a wider network of sensorimotor regions, which includes the supplementary motor area (SMA) and the cerebellum (11–13). In analogy to the above terminology, an interval may be defined as the temporal quantity between two successive markers. Interval timing tasks indicate that dopaminergic neurons show activity patterns, which consist of a burst at the beginning of a trial and a second burst at the expected time of reward, with sustained activity throughout the interval (14). Interval timing is typically conceptualized as a general activity that is inherent to the production and the perception of temporally structured behavior. As such, interval-based temporal processing is a crucial component of non-motor and motor aspects of activities as diverse as walking, speaking, or playing music, and, conversely the perception and the evaluation of the temporal structure that arises from the same activities (14). Due to the fundamental nature of basal ganglia contributions to sequencing and interval timing, the conceptual and structural overlap between these functions has widespread implications, especially if the patterning and chunking of motor and cognitive action sequences and temporal processing in cortico-striatal circuits reflect aspects of an even more general sensorimotor sequencing capacity, which guides production and perception. In the following, we will reflect on some of the potential consequences of this overlap in Parkinson’s disease (PD), focusing on the role of the basal ganglia and its interaction with associated temporal processing regions in this particular context.

## Parkinson’s Disease and (Dysfunctional) Temporal Processing

Impaired sequencing of motor actions is one of the hallmarks of PD, a neurodegenerative disease that leads to a loss of dopamine releasing neurons in the substantia nigra pars compacta of the basal ganglia. PD is commonly diagnosed on the basis of these primary motor symptoms, most characteristically the slowing of movements, rigidity, and resting tremor. However, PD is also a progressive disease, and these characteristic motor symptoms are asymmetric and may surface only after internal mechanisms fail to compensate for the impact of the disease (15). Although motor symptoms are most striking, PD typically also has a deteriorating impact on numerous cognitive functions that is apparent in the early “pre-motor” phase of the disease [for a recent review see Ref. (16)]. This most likely reflects the extensive structural and functional connectivity of the basal ganglia system. Consequently, non-motor symptoms can precede motor symptoms in early non-medicated patients (17).

In line with the rationale discussed above, damage to the basal ganglia system should also lead to impaired temporal processing as well as a temporally specific dysfunction of sequencing behavior. Moreover, the latter may be a consequence of the former as impaired temporal processing may factor into other motor and non-motor cognitive aspects of the disease, expressed in suboptimal timing in production and perception. The structural and functional characteristics and the interplay of brain regions that engage in temporal processing may therefore provide a novel perspective on some of the dynamic pathological and compensatory changes in regions such as the cerebellum and the SMA. Such changes have been observed in the progression of the disease but their specific role in the pathogenesis of PD remains unclear.

Seminal work in the temporal processing domain has established that the cerebellum is involved in precise and automatic discrete event-based (salient-feature) temporal processing, whereas the basal ganglia and associated cortico-striato-thalamo-cortical circuits engage in attention-dependent interval-based (continuous-event) temporal processing, thus creating an explicit representation of the temporal relation between successive events (12, 14, 18). However, apart from these primarily discussed systems, temporal processing most consistently activates the SMA and prefrontal regions (19). At least three important aspects should be emphasized: (i) these regions can engage in temporal processing also when very little or even no movement or movement preparation is involved (18), (ii) they can interact across different timescales, thereby potentially forming an integrative subcortico-cortical temporal processing network (10, 20), and, (iii) the same regions are affected by PD and change their activation patterns during the progression of the disease.

Parkinson’s disease patients display compromised performance in various temporal processing tasks spanning production and perception, which has been attributed to various components of a dysfunctional “internal clock” mechanism (21–23). Some aspects of this performance are reminiscent of primary motor symptoms. For example, PD patients tend to speed up during repetitive self-paced finger-tapping tasks, comparable to the typical phenomenon of gait festination (22, 24, 25). However, there is a considerable degree of heterogeneity in many results, which may reflect specific task characteristics, different stages of the disease, patient subgroups, as well as the differential engagement of compensatory mechanisms (26–28). With respect to compensation, it may be relevant to consider the relation of action selection, ordering, and implementation as separate from the specific temporal structuring of these processes as independent components of a general behavioral sequencing capacity. Thus, a better understanding of the role of other brain regions in temporal processing, most critically the cerebellum and the SMA, may pave the way to personalized and specifically targeted manipulations of temporal structure.

## Hypo- and Hyperactivity: Dysfunction or Compensation?

Post-mortem PD brain tissue analyses have revealed a selective loss of pyramidal neurons in the pre-SMA (29). However, neuroimaging studies that explicitly targeted the role of associated regions such as the cerebellum and the SMA in PD and also applied typical sequential temporal processing tasks are relatively rare. The existing evidence confirms complex patterns of interactions that change in the progression of the disease. Early studies observed that cerebellar hypoactivity in synchronization and continuation phases of a finger-tapping task at a base tempo of 600 ms that was partially normalized by medication (24). There are reports of hyperactivation of the pre-SMA in *de novo* PD patients relative to healthy controls, most likely reflecting the contribution of the pre-SMA to the temporal sequencing of self-initiated opening and closing movements of the (right) hand at approximately every 1000 ms (30). In the same study, this finding was paralleled by bilateral hyperactivation of the superior cerebellum (mainly ipsilateral) and hypoactivation of the ipsilateral inferior cerebellum. The authors hypothesized that these changes in the cerebellar activation pattern may reflect compensation for a dysfunctional cortico-striatal motor loop. Similarly, PD patients off levodopa medication showed hyperactivation of the left cerebellum and the SMA during a synchronization-continuation finger-tapping task using a base tempo of 750 ms. These results were also interpreted as an indication of compensation for cortico-striatal dysfunction (31). However, in another group of patients off medication, patterns of concurrent hypoactivation of the basal ganglia and the pre-SMA were observed during the performance of sequential hand movements (32), as well as of the basal ganglia and the cerebellum during a target interception task that required predictive motor timing (33). Other studies have shown hypoactivation of the pre-SMA and the caudal portion of the SMA paralleled by hyperactivation of the ipsilateral cerebellum in patients performing repetitive paced button presses (34), as well as an augmented recruitment of cerebello-thalamo-cortical circuits in PD patients performing continued finger tapping following a pacing sequence with a base tempo of 500 ms (35). While these examples are clearly selective, they serve to illustrate that the experimental tasks applied in PD research vary widely. The heterogeneity of the results reflects this variability, thus highlighting the need for individualized approaches, potentially on the basis of individual temporal processing profiles.

## Strategies for Intervention

Therefore, one goal could be to improve the efficiency and usability of interventions relying on rhythmic auditory cueing in order to provide patients with wearable devices that are customized to meet their individual therapeutic needs [for recent reviews, see Ref. (36–38)]. For example, a clearly distinguishable event structure in the hundreds-of-milliseconds range should be optimally suited to trigger automatic salient-feature cerebellar temporal processing mechanisms. Subliminal changes in this event structure could be used to compensate for impaired sequencing abilities due to basal ganglia pathology by pushing the dysfunctional system toward a more stable state over an extended period of time, e.g., by working against the tendency to accelerate movement rates. This strategy may assist or even circumvent the impaired build-up and evaluation of repetitive motor and cognitive action sequences in patients by assigning part of the sequencing task to potentially less affected brain regions such as the cerebellum. Accordingly, this perspective may shift the focus from basal ganglia pathology to the function of a distributed system, in which basal ganglia contributions to sequencing and temporal processing have to be interpreted relative to the function of the other network nodes. A potential starting point in this endeavor may be to obtain individual measures of basic temporal processing capacities such as “spontaneous motor tempo” (the ability to generate a temporally regular sequence of events) or “preferred perceptual tempo” (the preferred tempo of sensory events), which have been found to be correlated and linked to the ability to exploit temporal structure in a sensory task (39, 40). If combined, such basic measures may be indicative of some of the characteristics of the temporal processing network, i.e., the dysfunctional “internal clock” of a patient.

Due to the focus on the basal ganglia, cerebellar contributions to pathological and compensatory mechanisms are often overlooked (15). For example, although resting tremor is perhaps the most characteristic of the Parkinsonian symptoms, its origins are unknown. Research into this phenomenon has targeted multiple structures in the basal ganglia system, but it is of note that the tremor can be abolished via stimulation of thalamic targets of cerebellar output, which renders the cerebellum a potential source of the tremor-inducing pathological oscillations (15, 41, 42). Speculations of this kind also have to consider the progressive character of the disease, which may not only manifest in dynamic network changes but may also affect the causality underlying these assumptions. Overall, cerebellar compensatory mechanisms may be most efficient during the early stages of the disease, but they may fail once the pathological changes become more severe (15). However, the partial neglect of cerebellar contributions to PD also stands in contrast to the substantial evidence for reciprocal short-latency direct connections next to cortically mediated connections between the two systems (42–46). These structural connections suggest a tight coupling between the cerebellum and the basal ganglia and associated regions but their functional significance remains unclear. One candidate of particular interest in the context of sequencing and interval timing may be the cerebellar triggering of dopaminergic activity in prefrontal areas or the ventral tegmental area through projections via the thalamus that marks the beginning of a trial in interval timing tasks (11, 14, 47).

In addition to more systematic investigations of the cerebellum and the SMA in PD by means of temporal processing tasks that are known to activate these regions, the differentiation of their primary or compensatory engagement may be addressed on the basis of recent evidence for a more fine-grained structural differentiation of cerebellar, SMA, and also basal ganglia subregions. On the one hand, the distinct functional connectivity patterns revealed in this context may provide the opportunity to dissociate between primary pathological and secondary compensatory aspects and allow further exploration of specific network functions (5, 44, 48). On the other hand, these findings may be used to refine existing approaches, including the application of neurostimulation techniques such as repetitive transcranial magnetic stimulation (rTMS). rTMS has been used to target particular PD symptoms, patterns of hypoactivity and hyperactivity in specific regions, or fluctuations in behavior associated with long-term drug administration (49–53). For example, lateralization of the SMA and its differentiation into rostral and caudal subregions are reflected by different aspects of temporal processing tasks such as the temporal range or the engagement of sensorimotor as opposed to sensory processing (54, 55). Depending on the type and the temporal structure of the behavior of interest, such dissociations may be useful to identify the most promising target for the stimulation.

Task-dependent temporal processing characteristics may also partly explain the differential responses in specific regions to particular stimulation frequencies. For example, the right-lateralized SMA activity in temporal processing tasks in the suprasecond range may dominate the response to 1-Hz rTMS stimulation. 1-Hz stimulation has been found to impact the timing of anticipatory postural adjustments in PD patients if it is applied over the SMA but not over the dorsolateral premotor cortex, and to improve motor but not non-motor symptoms (56, 57). Obviously, further research is necessary to entertain these possibilities but the fundamental nature of temporal processing may bear the potential to improve the principal effectiveness of these methods, which are typically considered a promising form of treatment for PD (58, 59).

## Conclusion

Although the cerebellum and the SMA are affected in PD and should hence be considered in a more encompassing view of the disease, their contribution to the overall pathogenesis is a matter of debate. Perhaps most importantly, it is still unclear if changes in cerebellar and SMA activity are aspects of the primary pathology and/or secondary compensatory mechanisms (60). However, their contribution may be entirely secondary to the cell loss in the substantia nigra, which is the focus of basic research and therapeutic intervention. Further, a better understanding of their interaction with the basal ganglia deems necessary to account for the complexity of the disease and to open potential new directions for therapeutic interventions.

The overlap between the sequencing and the temporal processing functions ascribed to the basal ganglia and cortico-striatal circuits offer one such direction. Moreover, the relatively specific concepts that have been developed with respect to the neural mechanisms underlying temporal processing in the basal ganglia and the cerebellum may allow improvement of existing strategies such as cueing and stimulation techniques. In this context, knowledge about the interplay of the basal ganglia with other regions engaged in temporal processing may offer a means to improve behavioral compensatory strategies via the informed manipulation of temporal structure or the identification of promising targets for interventions targeting the neural level.

An interesting open question concerns the transfer of therapeutic effects from basic temporal processing tasks to more complex behavior or from motor to non-motor processing and vice versa (37). Such transfer effects may reflect the essentially sensorimotor nature of the overarching network, as well as the general behavioral function of the basal ganglia in the sequencing of actions in both domains. Accordingly, therapeutic intervention in PD may aim to balance the dysfunction of this overarching network by targeting specific network functions to improve performance in several domains rather than focusing only on the most prominent motor symptoms reflecting to an extent the increasing interest in non-motor features of the disease.

## Author Contributions

MS and SK have written the manuscript.

## Conflict of Interest Statement

The authors declare that the research was conducted in the absence of any commercial or financial relationships that could be construed as a potential conflict of interest.
